# Plant Growth-Promoting Rhizobacteria Improve Rice Response to Climate Change Conditions

**DOI:** 10.3390/plants12132532

**Published:** 2023-07-03

**Authors:** Susana Redondo-Gómez, Jennifer Mesa-Marín, Jesús A. Pérez-Romero, Vicente Mariscal, Fernando P. Molina-Heredia, Consolación Álvarez, Eloísa Pajuelo, Ignacio D. Rodríguez-Llorente, Enrique Mateos-Naranjo

**Affiliations:** 1 Departamento de Biología Vegetal y Ecología, Facultad de Biología, Universidad de Sevilla, 41012 Seville, Spain; jmesam@us.es (J.M.-M.); emana@us.es (E.M.-N.); 2 Departamento de Biología, Instituto Universitario de Investigación Marina (INMAR), Universidad de Cádiz, 11510 Puerto Real, Spain; jesusalperezromero@gmail.com; 3 Instituto de Bioquímica Vegetal y Fotosíntesis, cicCartuja, Universidad de Sevilla and CSIC, 41092 Seville, Spain; vicente.mariscal@ibvf.csic.es (V.M.); publio@us.es (F.P.M.-H.); consolacion@ibvf.csic.es (C.Á.); 4 Departamento de Microbiología y Parasitología, Facultad de Farmacia, Universidad de Sevilla, 41012 Seville, Spain; epajuelo@us.es (E.P.); irodri@us.es (I.D.R.-L.)

**Keywords:** bacterial consortium, efficiency of PSII photochemistry, elevated atmospheric CO_2_, gas exchange, inoculation, PGPR, temperature

## Abstract

Rice is one of the most important crops in the world and is considered a strategic crop for food security. Furthermore, the excessive use of chemical fertilizers to obtain high yields causes environmental problems. A sustainable alternative includes taking advantage of beneficial bacteria that promote plant growth. Here, we investigate the effect of five bacterial biofertilizers from halophytes on growth, and we investigate photosynthetic efficiency in rice plants grown under saline conditions (0 and 85 mmol L^−1^ NaCl) and future climate change scenarios, including increased CO_2_ concentrations and temperature (400/700 ppm and 25/+4 °C, respectively). Biofertilizers 1–4 increased growth by 9–64% in plants grown with and without salt in both CO_2_- temperature combinations, although there was no significant positive effect on the net photosynthetic rate of rice plants. In general, biofertilizer 1 was the most effective at 400 ppm CO_2_ and at 700 ppm CO_2_ +4 °C in the absence of salt. Inocula 1–5 also stimulated plant length at high CO_2_ levels without salt. Finally, the positive effect of biofertilization was attenuated in the plants grown under the interaction between salt and high CO_2_. This highlights the significance of studying biofertilization under stress interaction to establish the real potential of biofertilizers in the context of climate change conditions.

## 1. Introduction

Microorganisms are essential for the formation of soil structures in both natural and agricultural systems, and they are involved in fundamental processes such as the decomposition of organic matter and, in general, the cycle of C, N, P and S [1,2]. Some bacterial populations of the soil are capable of colonizing the rhizosphere or the interior of plants and stimulating growth, for which they are named plant growth-promoting rhizobacteria (PGPR) [2].

PGPR inoculants can carry out beneficial biological processes in agricultural systems with little or no negative impacts, as a natural solution [3]. This has led to a growing interest in developing PGPR-based biofertilizers for their application to different types of crops. PGPR promote plant growth through different mechanisms, such as [4,5,6,7] auxin production for root development (particularly indole-3-acetic acid, IAA), facilitation of nutrient uptake, atmospheric nitrogen fixation, siderophore production for iron uptake, phosphorus solubilization, synthesis of the enzyme ACC deaminase (cleaves ethylene, which is essential in response to stress), and biofilm production (which improves bacterial adhesion to root tissue and facilitation of nutrient uptake). Biofertilizers usually consist of PGPR consortia since a single strain often does not show all the mechanisms to promote host growth [5,8].

Rice (*Oryza sativa* L.) is an essential food product for the human population; it is estimated that world rice production for 2023 will be 517 million t [9]. Furthermore, rice is considered a strategic crop due to its wide distribution in soils and climates worldwide, as well as in a scenario of climate change [1,10]. However, studies that evaluate the effect of PGPR on rice growth are still limited [11,12,13,14]. Some of these studies have evaluated PGPR consortia or isolates not only as an alternative to chemical fertilization [1], but also to alleviate salinity [11,14] or drought stresses [12] in rice plants. This is relevant due to the fact that salt stress is limiting cultivation on lands around the world [15]. Anyway, none of these studies have considered the effect of PGPR on growth under the interaction of several abiotic factors. 

Studies assessing the effect of PGPR consortia on crops in combination with different abiotic stresses, such as salinity, temperature, and CO_2_ concentration, are very scarce. However, these studies are important for establishing the effectiveness of the use of biofertilizers in the context of the world’s changing climate [4,16]. In this context, halophytes are an excellent reservoir of halotolerant bacteria with plant growth-promoting traits that could be used for these studies [17]. 

Biofertilizers from halophytes have previously demonstrated their effectiveness in mitigating abiotic stress in different crops, including rice [4]. However, this previous study evaluated the effect of bacterial inoculants on growth, but not on the physiological response of crops. Therefore, our objective was to test the effects of five biofertilizers from halophytes on rice growth and physiological response under salinity stress (85 mM NaCl) since it has been previously shown that concentrations reduce the growth of rice [4], as do variations in atmospheric CO_2_ concentration and air temperature (400 ppm and 25/14 °C and 700 ppm +4 °C). 

## 2. Materials and Methods

### 2.1. Plant Materials, Growth Conditions, and Treatments

Rice seeds (*Oryza sativa* var. Puntal) were surface-disinfested in 0.5% (*w*/*v*) calcium hypochlorite for 20 min. Then, hypochlorite was removed by successive washing with sterilized tap water and germinated on a wet filter paper for 7 days. Germinated seedlings were transferred to 4 L closed tanks containing at least 20 plants, in +N BG110 medium [18]. Plants (*n* = 24) were grown in controlled-environment chambers at 400 ppm CO_2_ with a diurnal regime of 16 h of light at 25 °C and 8 h of darkness at 14 °C, 80% relative humidity and 300 μmolm^−2^ s^−1^ light flux (Aralab/Fitoclima 18.000 EH, Lisbon, Portugal). Treatments were stablished after 5 days of growth (see below).

Twenty-four different treatments were established (*n* = 20 per treatment): six biofertilization treatments (five rhizobacteria consortia + non-inoculated control), two salinity concentrations (0 and 85 mmol L^−1^ NaCl), and two CO_2_- temperature combinations—400 ppm CO_2_ at 25/14 °C (16/8 h) and 700 ppm CO_2_ at 29/18 °C (16/8 h). 

The salinity treatment was imposed by adding the appropriate concentration of salt (0 or 85 mmol L^−1^ NaCl) to the culture medium. This salt concentration was determined as the optimum for salt stress in rice in a previous analysis [4]. The conductivity of the tanks was monitored weekly with a conductivity meter (Probe GS3, Decagon, Pullman, WA, USA), and NaCl was added when necessary. The atmospheric CO_2_ concentrations in the chambers were continuously recorded by CO_2_ sensors (Aralab, Lisbon, Portugal) and maintained by supplying pure CO_2_ from a compressed gas cylinder (Air Liquide, B50 35 K). Rhizobacterial inoculation was carried out the day after setting environmental treatments (salinity, CO_2_ and temperature).

### 2.2. Consortia of Rhizobacteria

**Figure 1:**
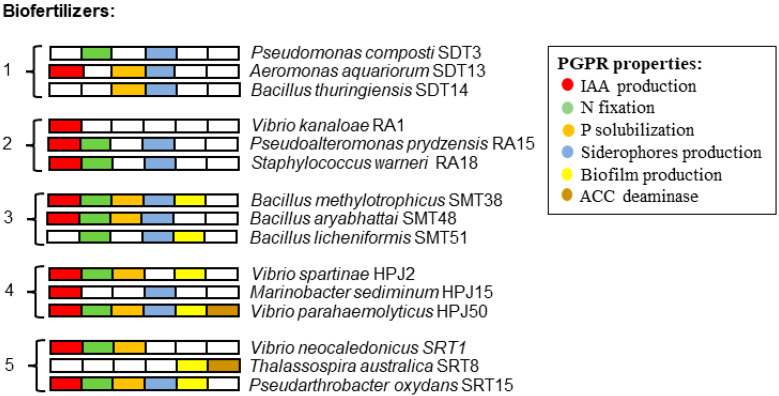
Plant growth-promoting rhizobacterial (PGPR) traits for the strains of biofertilizers used in this study. The rhizobacteria that compose biofertilizers were isolated from: 1, *Sporobolus montevidensis* (Arechav.) P.M. Peterson & Saarela; 2, *Allenrolfea occidentalis*; 3, *Sporobolus maritimus* (Curtis) P.M. Peterson & Saarela; 4, *Atriplex portulacoides*; and 5, *Salicornia europaea* (Information adapted with permission from Redondo-Gómez et al. [4]).

Five bacterial biofertilizers were used, which had been tested with eight crops: alfalfa, flax, maize, millet, wheat, strawberry, sunflower and rice [4]. They were constructed from rhizobacteria originally isolated from the rhizospheres of five different halophytes, commonly inhabiting salt marshes in southwestern Spain [16]. These five microbial consortia, containing three strains each, showed different PGPR activities likely promoting plant growth (see Figure 1). 

Bacterial suspension for inoculation was prepared as described previously [4]. In short, strains grown in TSB (Tryptone Soya Broth) medium were collected and resuspended in tap water to reach an OD_600_ of approximately 1.0 in order to produce a uniform bacterial concentration of all strains. The bacterial suspensions were mixed to produce the five final inoculant suspensions, as follows: strains SDT3, SDT13 and SDT14 were mixed to obtain Biofertilizer 1; strains RA1, RA15 and RA18 for Biofertilizer 2; strains SMT38, SMT48 and SMT51 for Biofertilizer 3; strains HPJ2, HPJ15 and HPJ50 for Biofertilizer 4 and strains SRT1, SRT8 and SRT15 were mixed in Biofertilizer 5. For plant inoculation, every 1.5 L pot was watered with 20 ml of the inoculant suspensions to get a final bacteria concentration of 10^5^ CFU/ml (estimating that a suspension of OD_600_ 1 corresponds to approximately 10^8^ CFU/ml).

### 2.3. Growth Measurements

After 20 d of growth in the different treatments, plant lengths (*n* = 20) were determined. Finally, the plants were harvested, and dry mass was determined after drying the samples at 80 °C for 48 h.

### 2.4. Gas Exchange

Gas exchange was measured in random tillers (*n* = 5) using an infrared gas analyser (LI-6400, LI-COR Inc., Lincoln, NE, USA; equipped with a light leaf chamber LI-6400-02B) in an open system one day before plant harvest. Net photosynthetic rate (A), stomatal conductance (Gs) and instantaneous water use efficiency (iWUE; ration between A and Gs) and intercellular CO_2_ concentration (Ci), were determined at a photon flux density (PPFD) of 1000 mmol photons m^−2^ s^−1^ (with 15% blue light to maximize stomatal aperture), a CO_2_ concentration surrounding the leaf of 400 mmol mol^−1^ air, an air temperature of 24 ± 1 °C, a relative humidity of 45 ± 5%, and a vapor pressure deficit of 2.0–3.0 kPa [19].

### 2.5. Chlorophyll Fluorescence

Chlorophyll fluorescence was measured in leaves (*n* = 12) from plants 20 d after treatment using a portable modulated fluorimeter (FMS-2; Hansatech Instruments Ltd., Kings Lynn, UK). Quantum efficiency of PSII photochemistry (Fv/Fm) was measured in leaves that were dark-adapted for 30 minutes using leaf clips designed for this purpose, and the maximum quantum efficiency of PSII photochemistry (Fv/Fm) was measured. Maximum efficiency of PSII was calculated as Fv/Fm (i.e., the quantum efficiency if all PSII centres were open) [20].

### 2.6. Statistical Analysis

Data were analysed using generalized linear models (GLMs). Statistical analysis was performed using the SPSS 26.0 statistical program (SPSS Inc., Chicago, IL, USA), using the Duncan test to establish the significance between treatments (*p* < 0.05). Before statistical analysis, to verify the assumptions of normality and homogeneity of the variances, Kolmogorov–Smirnov and Levene tests were used, respectively. 

## 3. Results 

### 3.1. Growth Measurements

**Figure 2:**
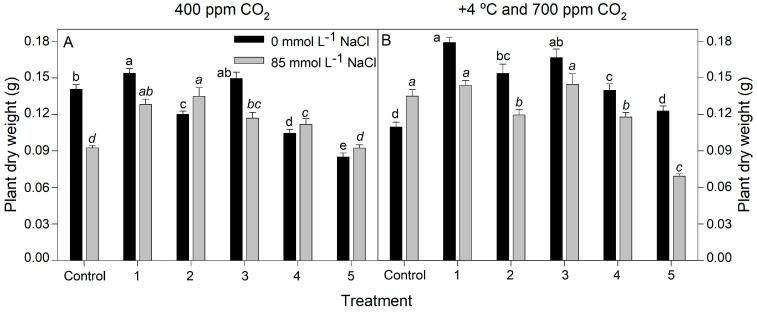
Dry weight of rice plants inoculated with rhizobacteria consortia, numbered 1 to 5 (control = non-inoculated plants), after 20 d of treatment at 400 ppm CO_2_ (**A**) and at +4 °C and 700 ppm CO_2_ (**B**) with 0 and 85 mmol L^−1^ NaCl. Each value represents the mean of twenty replicates ±SE. Different letters for each saline treatment (capital and italics letters for 0 and 85 mmol L^−1^ NaCl, respectively) indicate means that are significantly different from each other (GLM; Duncan test, *p* < 0.05).

**Figure 3:**
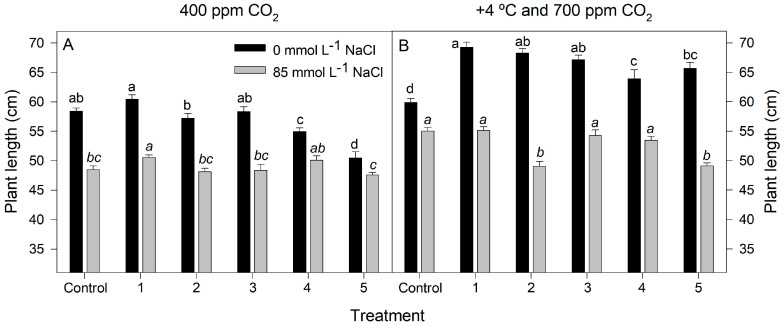
Length of rice plants inoculated with rhizobacteria consortia, numbered 1 to 5 (control = non-inoculated plants), after 20 d of treatment at 400 ppm CO_2_ (**A**) and at +4 °C and 700 ppm CO_2_ (**B**) with 0 and 85 mmol L^−1^ NaCl. Each value represents the mean of twenty replicates ±SE. Different letters for each saline treatment (capital and italics letters for 0 and 85 mmol L^−1^ NaCl, respectively) indicate means that are significantly different from each other (GLM; Duncan test, *p* < 0.05).

Inoculation with the different consortia of rhizobacteria had a significant effect on rice plants under different salinity conditions (0 and 85 mmol L^−1^ NaCl) at 400 ppm CO_2_ and 25 °C and 700 ppm CO_2_ +4 °C (GLM, *p* < 0.0001; Figure 2 and Figure 3). Bacterial inoculation increased plant growth at 400 ppm CO_2_ at 25 °C, regardless of saline treatment (*p* < 0.0001 for both with and without salt). Inoculum 1 showed the highest plant dry weight without salt compared to non-inoculated control plants by 9% (Figure 2A). Inocula 1–4 increased plant dry weight by 21–46% at 85 mmol L^−1^ NaCl and ambient CO_2_. 

Overall, biofertilization improved the growth of rice plants by 28–64% in the absence of salt at high CO_2_ and +4 °C (*p* < 0.0001). Nevertheless, there was not a significant effect from the dry weight of inoculated plants treated with 85 mmol L^−1^ NaCl (*p* < 0.0001; Figure 2B). In the same way, there was not a significant effect on the length increase in plants grown without salt at 400 ppm CO_2_ and 25 °C. However, in the presence of salt, plants treated with inoculum 1 showed an increased length by 4% with respect to the non-inoculated control (*p* < 0.0001; Figure 3A). 

Finally, when the two treatments were combined (85 mmol L^−1^ NaCl and high CO_2_ and +4 °C), there was no beneficial effect of biofertilization on the length of the plants. However, in the absence of salt, the plants treated with inocula 1–5 significantly increased in length compared to the non-inoculated control by 7–16% (*p* < 0.001; Figure 3B).

### 3.2. Gas Exchange

**Figure 4:**
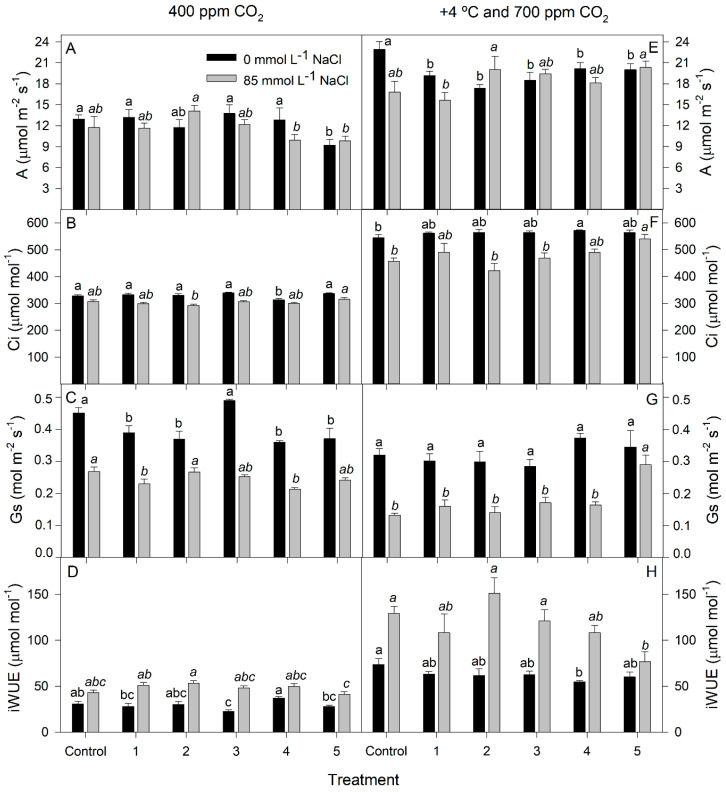
(**A**), Net photosynthetic rate (**A**,**E**); Ci, intercellular CO_2_ concentration (**B**,**F**); Gs, stomatal conductance (**C**,**G**); and iWUE, instantaneous water use efficiency (**D**,**H**) of rice plants inoculated with rhizobacteria consortia, numbered 1 to 5 (control = non-inoculated plants), after 20 d of treatment at 400 ppm CO_2_ (**A**–**D**) and at +4 °C and 700 ppm CO_2_ (**E**–**H**) with 0 and 85 mmol L^−1^ NaCl. Each value represents the mean of five replicates ±SE. Different letters for each saline treatment (capital and italics letters for 0 and 85 mmol L^−1^ NaCl, respectively) indicate means that are significantly different from each other (GLM; Duncan test, *p* < 0.05).

In general, the values of the net photosynthetic rate (A) were higher at elevated CO_2_ than those measured at ambient CO_2_ (GLM, *p* < 0.0001; Figure 4). There was no positive effect from biofertilization on A of plants grown in the absence of salt and ambient CO_2_; in fact, inoculum 5 had a negative effect on A (GLM, *p* < 0.0001; Figure 4A). Plants treated with biofertilizer 2 showed the highest A in the presence of salt, although there were no significant differences compared to control plants without inoculation (*p* <0.01). This A value corresponded to a lower intercellular CO_2_ concentration (Ci), but stomatal conductance (Gs) remained at values similar to those of the control (Figure 4B,C). In the same way, A values and inoculum 2 increased instantaneous water use efficiency (iWUE) by 23.5% at 85 mmol L^−1^ NaCl (Figure 4D).

The trend described previously was also observed at 700 ppm CO_2_ at +4 °C. Only in the presence of salt there was a beneficial effect from inoculation (GLM, *p* < 0.0001). Again, inoculum 2 improved A values by 19% compared to non-inoculated control plants (Figure 4E). This increase also corresponded to lower Ci values, while Gs remained unchanged with respect to the control (Figure 4F,G). Finally, plants treated with biofertilizer 2 showed the highest iWUE values (Figure 4H).

### 3.3. Chlorophyll Fluorescence

**Figure 5:**
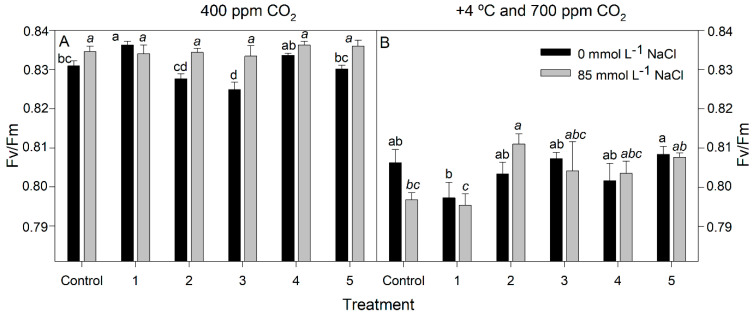
Maximum quantum efficiency of PSII photochemistry (Fv/Fm) of rice plants inoculated with rhizobacteria consortia, numbered 1 to 5 (control = non-inoculated plants), after 20 d of treatment at 400 ppm CO_2_ (**A**) and at +4 °C and 700 ppm CO_2_ (**B**) with 0 and 85 mmol L^−1^ NaCl. Each value represents the mean of ten replicates ±SE. Different letters for each saline treatment (capital and italics letters for 0 and 85 mmol L^−1^ NaCl, respectively) indicate means that are significantly different from each other (GLM; Duncan test, *p* < 0.05).

Biofertilizer 1 was the only inoculant that produced a beneficial effect on photosystem II (PSII) activity in the rice plants grown at 400 ppm CO_2_ and 25 °C since the maximum quantum efficiency of PSII photochemistry (Fv/Fm) of plants treated with this inoculum was significantly higher than the non-inoculated control at 0 mmol L^−1^ NaCl (*p* < 0.0001). This effect was not observed in the presence of salt (*p* > 0.05; Figure 5A) and was reversed at 700 ppm CO_2_ +4 °C, having only a significant positive effect on Fv/Fm values in the presence of salt. Notably, inoculum 2 was the one producing an improvement PSII activity, recording a higher Fv/Fm value than the control (*p* < 0.05; Figure 5B). Finally, Fv/Fm values were lower at elevated CO_2_ +4 °C than at ambient CO_2_, regardless of saline treatment (*p* < 0.0001).

## 4. Discussion

Both salinity and 700 ppm CO_2_ +4 °C treatments were stressful conditions for rice plants, as they reduced plant growth and increased photoinhibition, that is, low Fv/Fm values. Our results are in agreement with previous results found for the Shiroudi rice variety, which reduced its total dry weight at 700 ppm CO_2_ compared to plants treated with 360 ppm CO_2_ [21]. Nonetheless, Cheng et al. [22] found that dry weight was higher for rice plants grown at high CO_2_ concentration and high or low night temperatures (32 and 22 °C, respectively). In the same way, Feng et al. [23] observed a greater relative growth rate in rice seedlings at 700 ppm CO_2_ and 27 °C than those grown at 450 ppm CO_2_ and 23 °C, and this trend was maintained in the presence of 1.1 g Na L^−1^. This is in line with our findings in the presence of salt for non-inoculated plants. We have reported an increase in plant dry weight at 700 ppm CO_2_ +4 °C and 85 mmol L^−1^ NaCl with respect to plants at ambient CO_2_. This was not the case for the plant length since the lowest size was recorded in plants grown under the interaction of the two stressors. On the other hand, Kazemi et al. [21] concluded that the negative effects of salinity on rice plant growth were intensified by elevated CO_2_ conditions, which enhanced cell membrane damage. The adverse effects of salinity are also intensified with increasing temperature [24].

Interestingly, the higher rate of net photosynthesis measured for the non-inoculated control at elevated CO_2_ +4 °C did not correspond to higher dry weight with respect to the non-inoculated control at ambient CO_2_. Carbon dioxide is expected to increase photosynthesis, dry matter production, and yield; decrease stomatal conductance rate and transpiration in C3 and C4 species; and improve the water use efficiency of plants [25]. However, high CO_2_ suppresses photorespiration, which has been linked to nitrogen uptake capacity [26]. It has been reported that *Arabidopsis* mutants with impaired nitrate uptake capacity showed a greater effect on growth at elevated CO_2_ concentrations than at lower concentrations [27], which was due to a reduction in the mutant’s ability to assimilate nitrate at elevated CO_2_. In our study, in the absence of salt and at 700 ppm CO_2_ +4 °C, the effect on growth might be overall counteracted by biofertilization, as all consortia contained strains with nitrogen fixation capacity. Furthermore, photorespiration plays an important role in plant carbon metabolism and provides tolerance to stress in plants [28]. Photorespiration counteracts photoinhibition and reactive oxygen species (ROS) production under high light conditions [29,30], which cause lipid peroxidation and chlorophyll degradation [31]. In this way, we found lower Fv/Fm values, at 700 ppm CO_2_ +4 °C with respect to ambient CO_2_.

In the absence of stress, biofertilizer 1 was the only one stimulating the growth of the rice plants. In contrast, biofertilizers 1–4 increased rice growth in the presence of stressors, salinity, or high CO_2_ +4 °C. Finally, no effect from biofertilization was registered in the plants grown under the stress interaction, high CO_2_ +4 °C and 85 mmol L^−1^. A previous study found that inoculation with *Bacillus pumulis*, *Pseudomonas pseudoalcaligenes* alone and in combination increased the plant dry weight of the GJ-17 rice variety in the presence and in the absence of salt [32]. In this way, biofertilizer 1 was the only one that contained a strain of the genus *Pseudomonas*. Redondo-Gómez et al. [4] already described that consortia obtained from halophytes were really useful for salt stress alleviation under stress conditions [16]. 

The present work highlights the use of microbial consortia from halophytes to alleviate plant growth under a combination of abiotic stresses, even if a low effect on the improvement of photosynthetic rate is observed. Nevertheless, there was no determining property in the inocula, but rather in the combination of them. This makes the use of a microbial consortium more useful than the use of independent strains [5,8]. Biofertilizers 3 and 4 are composed of two PGPR strains with the ability to produce biofilm, and therefore, they are capable of chelating different cations. In this way, bacteria can bind with the Na+ ion under salt stress by means of the secretion of exopolysaccharides (EPS), consequently reducing its toxicity in the soil [33]. However, these biofertilizers were not the most effective in stimulating plant growth at 400 ppm CO_2_ and 85 mmol L^−1^ NaCl; it was consortium 2 that exhibited greater IAA production than consortia 1, 3 or 5. Higher IAA production could stimulate root growth and improve the uptake of nutrients and water for the plant. The flow of water and nutrients has previously been reported to stabilize stomatal conductance and transpiration rate, improving photosynthetic rate, iWUE, and starch production, and therefore, stimulating plant growth under salinity conditions [13]. Indeed, we also observed higher iWUE values for plants treated with salt and biofertilizer 2 at ambient CO_2_. Interestingly, biofertilizer 1, with a lower IAA production capacity, stimulated the growth of rice plants to the same extent as biofertilizer 2, although the first contained PGPR capable of solubilizing P and had greater siderophore production, which would also imply an improvement in the nutritional status of the plants. Finally, biofertilizer 5, which along with 4 was the most complete in terms of PGPR traits, only improved plant length at high CO_2_ levels without salt.

## 5. Conclusions

Biofertilizers containing microbial consortia from halophytes proved to be effective in mitigating the negative effects of salinity and high CO_2_ concentration and temperature on rice plants, although they did not show an effect when the interaction between these abiotic factors was tested. These biofertilizers improved plant growth and physiological response. Biofertilizers 1–4 stimulated plant growth in the presence of salt stress or high CO_2_ +4 °C. Furthermore, these consortia kept photosynthetic rate, instantaneous water use efficiency, and the maximum quantum efficiency of PSII photochemistry of the rice plants at similar values to those of the control. The combination of strains in a consortium shows a synergistic effect that provides better PGPR features than the individual strains. 

Studies that identify the effect of different stressors on crop are very scarce, and studies that consider inoculation with PGPR to mitigate the effects of these stressors are even more so. This highlights the significance of developing studies that consider stress interaction to establish the real potential of biofertilizers in the context of climate change conditions.

Finally, it would be necessary to analyse in the future biofertilizer effectiveness in field experiments and study their effect on crop production.

## Data Availability

The data presented in this study are available on request from the corresponding author.
